# Aryl hydrocarbon receptor is a prognostic biomarker and is correlated with immune responses in cervical cancer

**DOI:** 10.1080/21655979.2021.2006953

**Published:** 2021-12-25

**Authors:** Jiasui Wang, Yilidana Mijiti, Yalin Chen, Zaoling Liu

**Affiliations:** a School of Public Health, Xinjiang Medical University, Urumqi, China; b The First Affiliated Hospital of Xinjiang Medical University, Urumqi, China; c The Sixth Division Hospital of Xinjiang Production and Construction Corps, China

**Keywords:** AHR, cervical cancer, prognosis, tumor-infiltrating, bioinformatics

## Abstract

Aryl hydrocarbon receptor (AHR) plays an important role in tumor development. However, its function in cervical cancer has not been fully elucidated. We evaluated the ten genes that are predicted to associate with AHR protein interaction. The comprehensive scores were: CYP1A1, ARNT2, HSP90AA1, ARNT, AIP, PTGES3, HSP90AB1, CYP1B1, ESR1, MAF, respectively. In addition, we showed that levels of AHR and its related genes were correlated with the immune infiltration and expression of immuno-regulators (immunoinhibitors, immunostimulators, MHC molecules) levels in cervical cancer. High expression of AHR, CYP1A1, HSP90AA1, and HSP90AB1 and low expression of ESR1 were negatively correlated with the prognoses of cervical cancer patients. The Cox multivariate regression showed that high expression of AHR (*HR *= 1.874, *95% CI *= 1.069–3.285, *P*= 0.028) and CYP1A1 (*HR *= 1.822, *95%CI *= 1.077–3.080, *P*= 0.025) were risk factors for prognosis in patients with cervical cancer. IHC results indicated that AHR and CYP1A1 were widely expressed in cervical cancer. These findings suggest that AHR and CYP1A1 may serve as prognostic biomarkers for determining prognosis and immune infiltration in cervical cancer.

## Introduction

Cervical cancer is a common malignancy among women worldwide. According to the 2020 global cancer report, there were 570,000 new cases of cervical cancer, and 311,000 deaths in 2018 [1]. Therefore, it is important to study the potential mechanisms and biomarkers involved in cervical cancer. Aryl hydrocarbon receptor (AHR) is a ligand-activated transcription factor that, can be activated by a variety of chemicals. AHR was originally investigated as a receptor of toxic substances in environmental pollution [2]. Later studies showed that AHR plays an important role in the occurrence and development of various types of cancer [3]. AHR is overexpressed in breast cancer, skin cancer, lung cancer, and other tumor tissues [4,5], suggesting that AHR is a potential target gene for the treatment of malignant tumors. However, there has not been much research have investigated the role of AHR and in cervical cancer.

Disorders of the immune response system in the tumor microenvironment are involved in the occurrence and development of cervical cancer [6]. AHR is closely related to innate immunity and adaptive immunity [7]. Inhibitory pathways related to AHR and its metabolites may be activated in the tumor microenvironment, thus promoting immune escape and tumor progression [8]. In addition, AHR is involved in various immune cell regulation processes. The presence of endogenous AHR ligands may promote the development of T cells in the tumor microenvironment. AHR signal transduction in CD4 + T cells can promote the differentiation of CD4 + and CD8 + cell into adaptive T cells [9]. AHR regulates the differentiation of B cells by inhibiting the transcription of EBF1 and PAX5 [10]. AHR is also a key cofactor involved in the production of IL-10 by NK cells [11]. Furthermore, the study has shown that AHR is a transcription factor that determines the differentiation of monocytes of mice, and that the activation of AHR promotes the differentiation of monocytes into dendritic cells and interferes with the differentiation of monocytes into macrophages [12]. The level of immune cell infiltration in the tumor microenvironment is closely related to the survival and prognosis of patients [13]. Wang et al. [14] evaluated the landscape of tumor infiltrating immune cells as prognostic biomarkers in cervical cancer, which suggested that the level of tumor infiltrating immune cells is a decisive factor in the prognosis of cervical cancer. However, the mechanisms underlying the role of AHR in tumor progression and tumor immunology in cervical cancer remain unclear.

In the present study, we propose the hypothesis that AHR may play an important role in the occurrence and development of cervical cancer. To investigate the immune expression and clinical significance of AHR and related genes in cervical cancer. We analyzed the expression of AHR and related genes and its correlation with immune responses, and prognosis in cervical cancer using the Gene Expression Omnibus (GEO), and the Tumor Genome Atlas (TCGA) database. We used immunohistochemistry to verify the expression of AHR in cervical cancer. The results indicated that the AHR may be a potential immunotherapy target and biomarker for cervical cancer.

## Materials and methods

### Data collection

The transcriptome expression data sets of cervical cancer were obtained from the GEO databases (https://www.ncbi.nlm.nih.gov/geo/): GSE7410 (45 cases), GSE 67522 (42 cases), and GSE 6791 (28 cases), and the TCGA database (https://portal.gdc.cancer.gov/): 309 cases.

### LinkedOmics database analysis

Co-expression genes of AHR were identified and gene set enrichment analysis in cervical cancer was performed using the LinkedOmics database (http://linkedomics.org/login.php) [15]. The threshold was determined according to the following values: *P* < 0.05, *FDR* < 0.05.

### STRING database analysis

The AHR protein-protein interaction network was searched using the STRING database (https://string-db.org/) [16], the ten protein coding genes that are predicted to associate with AHR were extracted for subsequent analysis.

### Differential expression analysis

GEO2R (https://www.ncbi.nlm.nih.gov/geo/geo2r/) tool was used to evaluate the differential expression of AHR and related genes in tumors and normal tissues (GSE7410, GSE67522, GSE6791).

### TIMER database analysis

TIMER is a comprehensive resource for the systematic analysis of immune infiltrates across in diverse cancer types (https://cistrome.shinyapps.io/timer/) [17]. The TIMER database includes 10,897 samples from 32 cancer types from TCGA to estimate the abundance of immune infiltrates. We analyzed the correlation between the expression of AHR and related genes and the abundance of immune infiltrates, including B cells, CD4 + T cells, CD8 + T cells, neutrophils, macrophages, and dendritic cells, via gene modules.

### TISIDB database analysis

The correlations between the expression of AHR and related genes and immuno regulators (including immunoinhibitors, immunostimulators, and MHC molecules) were calculated using TISIDB database (http://cis.hku.hk/TISIDB/) [18].

### Survival analysis

The correlation between the expression of AHR and related genes and survival in cervical cancers was analyzed using Kaplan-Meier plotter (http://kmplot. com/analysis/) [19]. The hazard ratio (HR) with 95% confidence interval and log-rank *P*-value were computed. The Cox regression model was used to explore the prognostic factors of patients with cervical cancer, using the forward step method (inclusion: *P*< 0.05, exclusion *P*> 0.1), *P*< 0.05 was considered statistically significant.

### Immunohistochemistry analysis

Cervical paraffin tissue samples were obtained from the Sixth Division Hospital of Wujiaqu City, Xinjiang Uygur Autonomous Region. For immunohistochemical analysis, cervical sections were de-paraffinized with xylene, and dehydrated in a graded ethanol series. Endogenous peroxidase activity was eliminated with 3% H_2_O_2_-methanol-PBS (0.01 M, pH 7.2) for 20 min, and sections were then washed three times (every 5 min) in phosphate-buffered saline (PBS). The sections were boiled in 0.01 mol/L trisodium citrate buffer (pH 6.0) to retrieve the antigens, then allowed to cool. The sections were incubated overnight at 4°C with the relevant primary rabbit antirat polyclonal antibody. The primary antibodies included AHR and CYP1A1 (Beijing Bioss Biotechnology Co., Ltd, diluted 1:100). The sections were washed in PBS. The second antibody (Zhongshan Jinqiao, Beijing) was incubated at 37°C for 25 min, and overlaid with DAB Kit (Zhongshan Jinqiao, Beijing). Negative controls were obtained by omitting the primary antibodies. Sections were counterstained with hematoxylin. The images were acquired at 40× magnification using an optical microscope (Leica, Leica Corporation, German). The positive expression rate of AHR was calculated by Image J software. This study was approved by the ethics committee of the First Affiliated Hospital of Xinjiang Medical University (Number: K202103-19).

### Statistical analysis

R 4.0.5 was used for statistical analysis. The ‘survival’ and ‘RMS’ package was used to draw the Nomogram. The difference in expression of AHR between cervical tissue and cervical cancer tissue was compared by the χ^2^ test.

## Results

AHR and related genes may be closely associated with cervical cancer. Therefore, we investigated the immune response of these genes in cervical cancer and their relationship with prognosis. We found that AHR and its related genes associated significantly with immune response and prognosis.

## AHR and co-expressed genes are involved in biological regulation in cervical cancer

**Figure 1.**
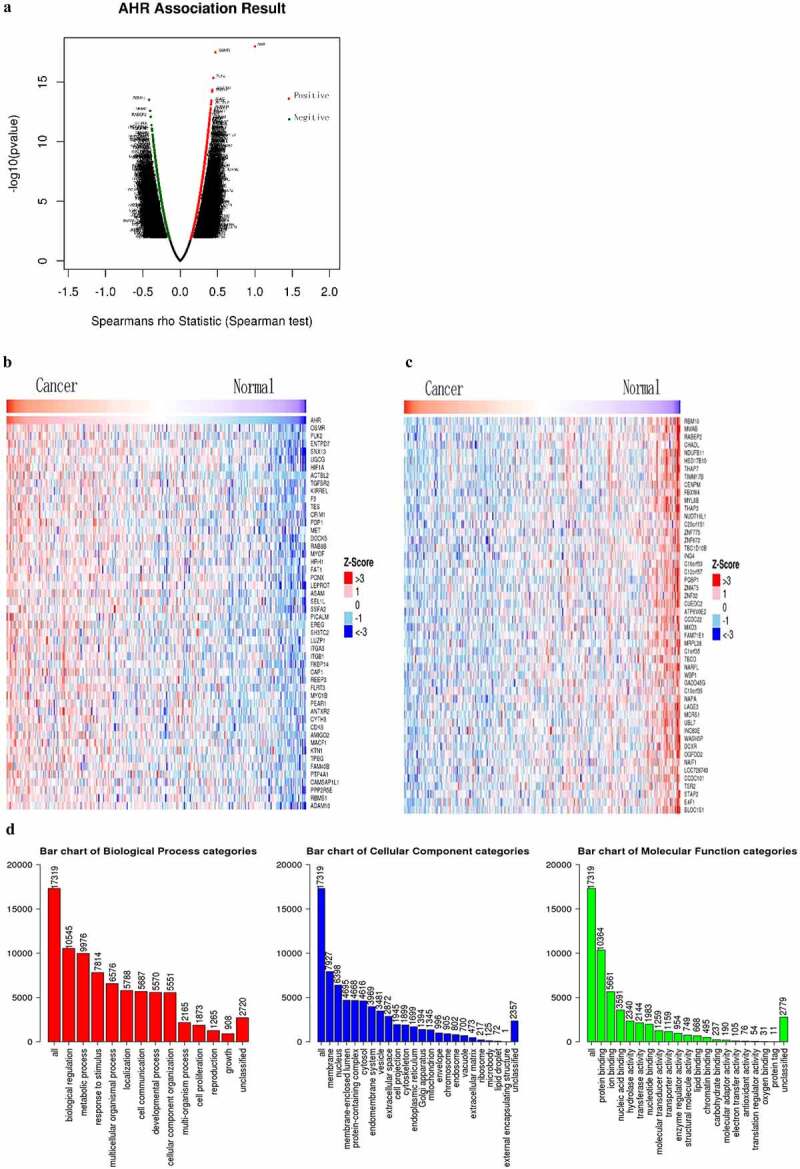
The relationship between AHR and its associations and gene set enrichment analysis in cervical cancer based on the LinkedOmics database. Map of AHR-related genes(a). Heat map of the top 50 genes positively (b) and negatively (c) correlated with AHR. Gene set enrichment analysis of AHR (d)

The relationship between the expression of AHR and related genes and biological regulation in cervical cancer was analyzed using the LinkedOmics database. As shown in Figures 1, 3,209 genes were positively correlated with AHR and 2,651 genes were negatively correlated (*P* < 0.05, *FDR* < 0.05) (Figure 1a). The top 50 genes positively and negatively correlated with AHR are shown in a thermogram in Figure 1b-c. Gene set enrichment analysis showed that AHR-related genes were distributed in the cell membrane and nucleus (Figure 1d), and were involved in biological regulation, metabolic processes, stimulation response, and multicellular biological processes. The main molecular functions included binding protein, ions and nucleic acids.

**Figure 2.**
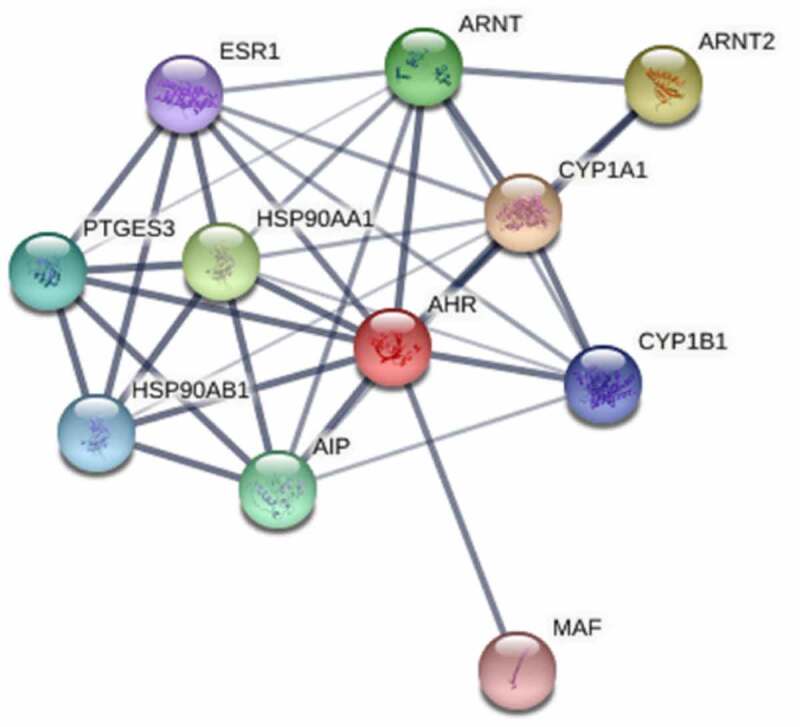
Interactions between AHR and related proteins

**Figure 3.**
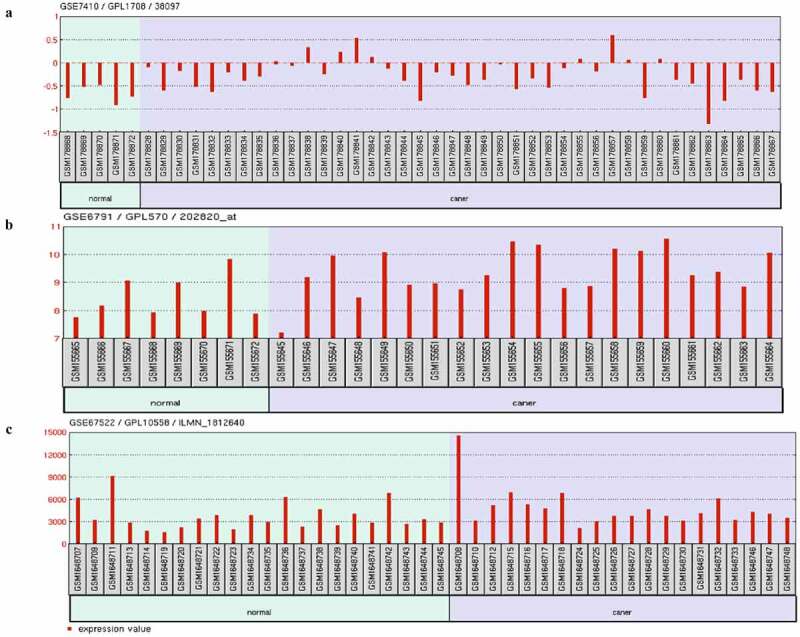
Expression of AHR in GEO cervical cancer datasets. Expression of AHR in GSE7410(a), GSE67522(b)and GSE6791(c)

## Protein interaction comprehensive score of AHR with its related genes

In order to further narrow the range of genes related to AHR. We extracted 10 genes that are predicted to associated with AHR from the STRING database. And we calculated the protein interaction score of AHR with its related genes, 10 genes: CYP1A1, ARNT2, HSP90AA1, AIP, PTGES3, HSP90AB1, CYP1B1, ESR1, and MAF (Table 1, Figure 2).

**Table 1.** The interaction scores of AHR with its related proteins

Gene	Annotation	Scores
CYP1A1	Cytochrome P450 1A1; Cytochromes P450 are a group of heme-thiolate monooxygenases. In liver microsomes	0.997
ARNT2	Aryl hydrocarbon receptor nuclear translocator 2; Transcription factor that plays a role in the development of the hypothalamo-pituitary axis	0.989
HSP90AA1	Heat shock protein HSP 90-alpha; Molecular chaperone that promotes the maturation	0.989
ARNT	Aryl hydrocarbon receptor nuclear translocator; Required for activity of the Ah (dioxin) receptor. This protein is required for the ligand-binding subunit to translocate from the cytosol to the nucleus after ligand binding.	0.989
AIP	AH receptor-interacting protein; May play a positive role in AHR-mediated (aromatic hydrocarbon receptor) signaling	0.985
PTGES3	Prostaglandin E synthase 3; Cytosolic prostaglandin synthase that catalyzes the oxidoreduction of prostaglandin endoperoxide H2 (PGH2) to prostaglandin E2 (PGE2).	0.974
HSP90AB1	Heat shock protein HSP 90-beta; Molecular chaperone that promotes the maturation	0.973
CYP1B1	Cytochrome P450 1B1; Cytochromes P450 are a group of heme-thiolate monooxygenases. In liver microsomes	0.943
ESR1	Estrogen receptor; Nuclear hormone receptor. The steroid hormones and their receptors are involved in the regulation of eukaryotic gene expression and affect cellular proliferation and differentiation in target tissues.	0.888
MAF	Transcription factor Maf; Acts as a transcriptional activator or repressor. Activates the expression of IL4 in T helper 2 (Th2) cells. Increases T-cell susceptibility to apoptosis by interacting with MYB and decreasing BCL2 expression.	0.871

## Expression of AHR and related genes in GEO datasets

Three cervical cancer transcriptome datasets (GSE7410, GSE67522, and GSE6791) from the GEO database were selected, including 80 cases of cervical cancer and 35 cases of normal. The results showed that the expression of AHR in all data sets was statistically significantly different (*P*< 0.05), except AIP; the expression of nine other genes related to the AHR gene in at least one dataset was different in Table 2(*P* < 0.05). The mRNA expression of AHR was lower in the normal group than in the cervical cancer group (Figure 3).

**Table 2.** The expression of AHR and related genes in GEO data sets

Gene	GSE7410			GSE67522			GSE6791	
	*P*	*logFC*		*P*	*logFC*		*P*	*logFC*
AHR	**0.0204**	−0.4101		**0.0439**	−0.3850		**0.0081**	−0.9289
CYP1A1	**<0.001**	1.1445		0.3300	0.0638		**<0.001**	0.5909
ARNT2	**0.0027**	0.5703		0.7710	0.0780		0.4460	−0.2506
HSP90AA1	**0.0002**	−0.8569		**<0.001**	−0.6930		**<0.001**	−1.8095
ARNT	**<0.001**	0.4053		0.2770	0.2080		**<0.001**	−1.0398
AIP	0.0863	−0.2581		0.2390	−0.2130		0.0959	−0.2656
PTGES3	**<0.001**	−0.7485		0.8230	−0.0369		**<0.001**	−1.8415
HSP90AB1	**0.0010**	−0.4403		0.4780	−0.1330		**0.0001**	−1.4151
CYP1B1	**<0.001**	1.5338		**0.0005**	1.5100		0.7630	−0.1137
ESR1	**<0.001**	2.2344		-	-		**0.0464**	0.2908
MAF	**<0.001**	0.9239		**0.0003**	1.3100		0.8690	0.0755

## The expression of AHR and related genes is correlated with immune infiltration level in cervical cancers

**Figure 4.**
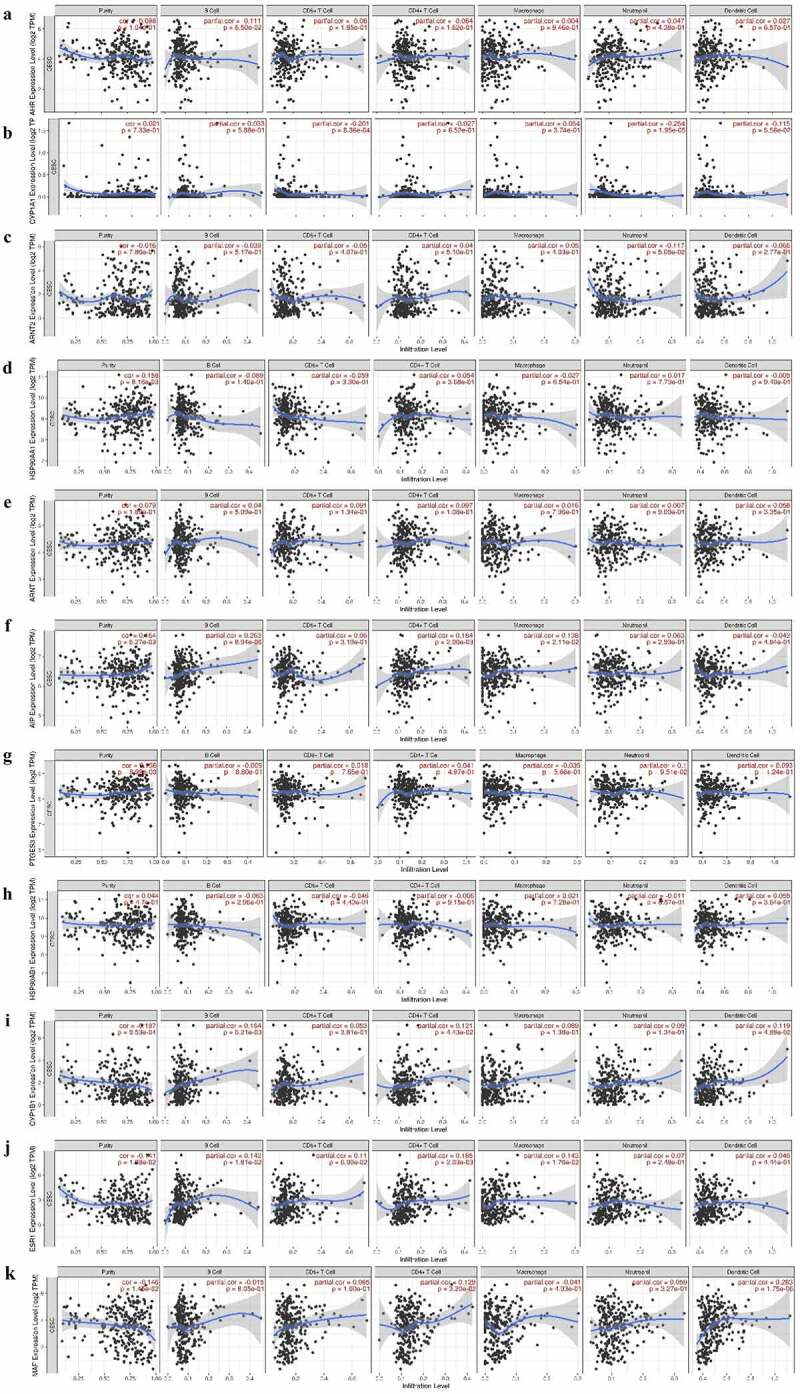
The expression of AHR and related genes was correlated with immune infiltration level in cervical cancer based on TIMER database. AHR(a), CYP1A1(b), ARNT2(c), HSP90AA1(d), ARNT(e), AIP(f), PTGES3(g), HSP90AB1(h), CYP1B1(i), ESR1(j)and MAF(k) with cervical cancer associated tumor sample purity, B cells, CD8 + T cells, CD4 + T cells, macrophages, neutrophils, and dendritic cells

We investigated whether AHR and its related genes were correlated with immune infiltration levels in cervical cancer. According to the TIMER database (Figure 4), CYP1A1 (Figure 4b) was negatively correlated with CD8 + T cells (*r*= −0.201, *P*= 8.36e^−04^) and neutrophils (*r*= −0.253, *P*= 1.95e^−05^). HSP90AA1 (Figure 4d) was positively associated with tumor purity (*r*= 0.158, *P*= 8.16e^−03^). AIP (figure 4f) was positively correlated with tumor purity (*r* = 0.164, *P* = 6.27e^−03^), B cells (*r* = 0.2633, *P*= 8.94e^−06^), CD4 + T cells (*r* = 0.184, *P* = 2.06e^−03^) and macrophages (*r* = 0.138, *P*= 0.05). PTGES3 (Figure 4g) was positively correlated with tumor purity (r = 0.156, *P* = 8.99e^−03^). CYP1B1(Figure 4i) was significantly associated with tumor purity (*r*= −0.197, *P* = 9.53e^−04^), B cells (*r* = 0.164, *P*= 3.81e^−03^), CD4 + T cells (*r* = 0.121, *P* = 4.43e^−02^), and dendritic cells (*r*= 0.119, *P* = 4.88e^−02^). ESR1(Figure 4j) expression level was negatively associated with tumor purity (*r*= – 0.141, *P*= 1.88e^−02^), B cells (*r* = 0.141, *P*= 1.81e^−02^), CD4 + T cells (*r* = 0.185, *P*= 2.03e^−03^), and macrophages (*r* = 0.143, *P* = 1.76e^−02^). MAF (Figure 4k) was significantly associated with CD4 + T cells (*r*= 0.129, *P*= 3.20e^−02^) and dendritic cells (*r* = 0.283, *P*= 1.75e^−06^), and negatively correlated with tumor purity (*r* = −0.146, *P* = 1.45e^−02^).

## The expression of AHR-related genes is correlated with immuno regulators in cervical cancers

**Figure 5.**
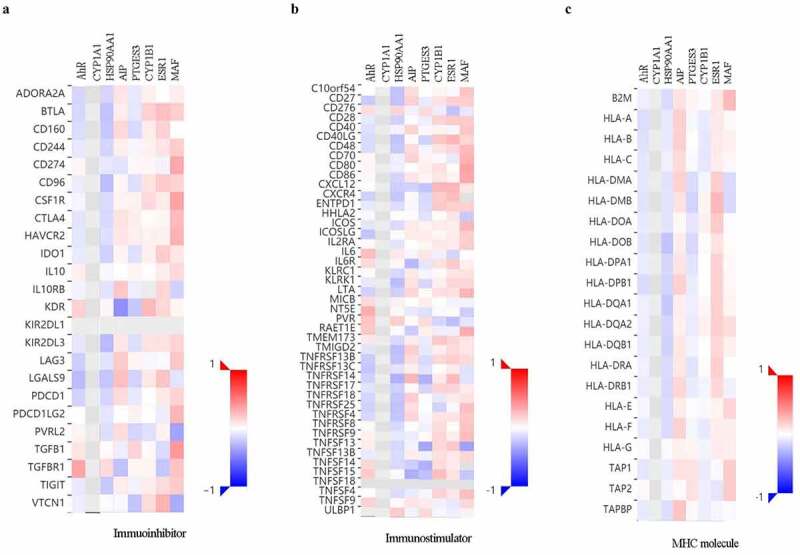
Correlations between AHR and related genes and immuno regulators in cervical cancer based on TISIDB database. The correlations between the expression of AHR, CYP1A1, HSP90AA1, PTGES3, CYP1B1, ESR1, and MAF and immunoinhibitors (a), immunostimulator (b), and MHC molecules (c) were calculated based on TISIDB database

To further explore the effects of AHR and related genes on immune responses, the correlation between the expression of AHR and related genes and immune regulators was analyzed (Figure 5). The results indicated that AHR, HSP90AA1, and PTGES3 were negatively correlated, whereas ESR1 and MAF were positively correlated with immunoinhibitors, immunostimulators, and MHC molecules.

## High AHR and CYP1A1 expression impacts the prognoses of cervical cancer patients

**Figure 6.**
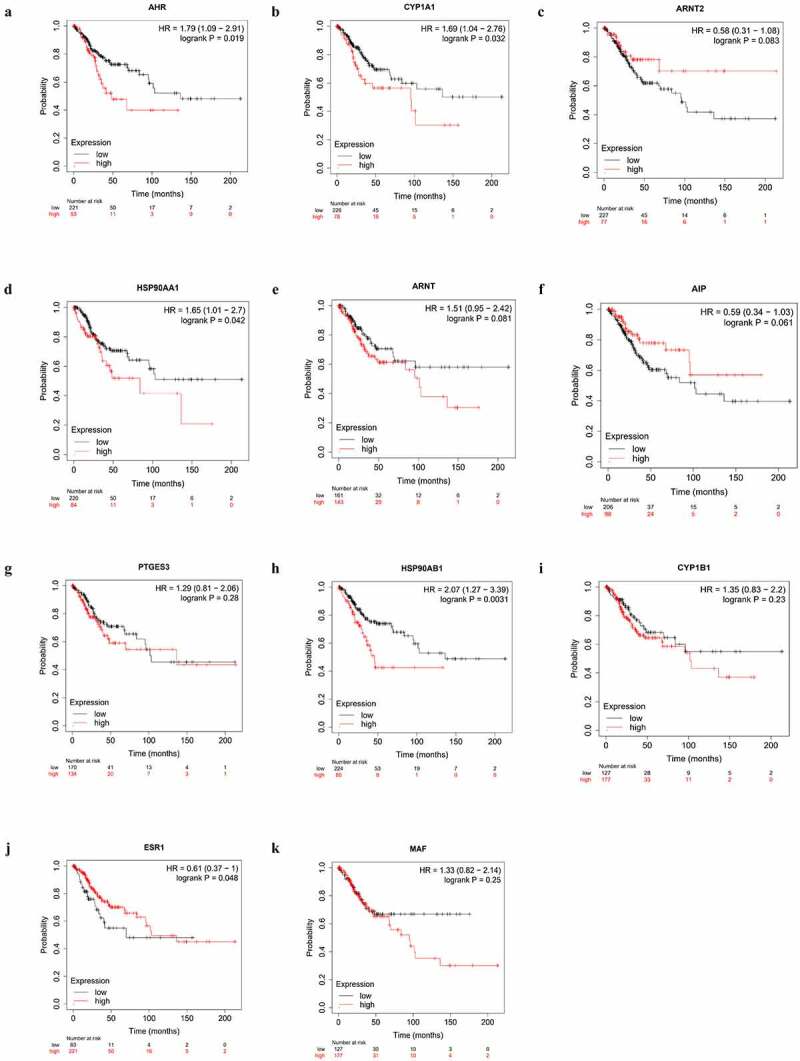
Alterations of AHR(a), CYP1A1(b), ARNT2(c), HSP90AA1(d), ARNT(e), AIP(f), PTGES3(g), HSP90AB1(h), CYP1B1(i), ESR1(j), MAF(k) were correlated with prognosis in cervical cancer patients based on the Kaplan-Meier database

**Figure 7.**
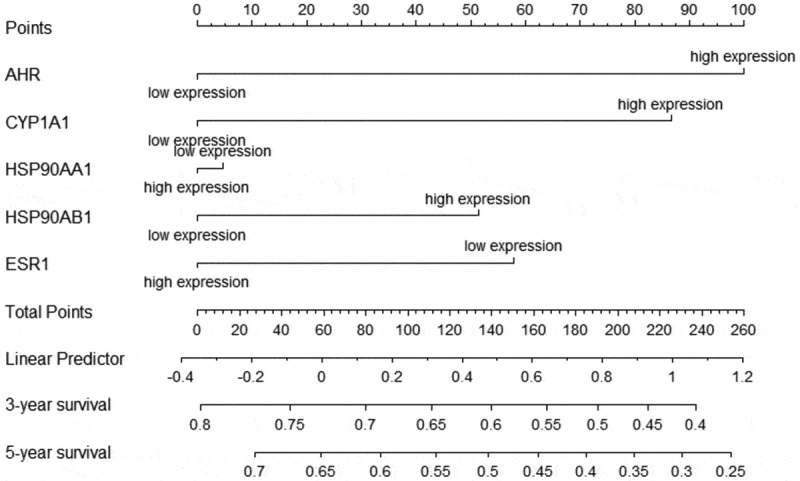
Nomogram of factors associated with survival time in cervical cancer patients

We investigated the relationship between the expression of AHR and related gene and the prognosis cervical cancer patients using the Kaplan-Meier plotter. Overexpression of AHR was associated with worse prognosis in cervical cancer patients (Figure 6a). Specifically, the median overall survival time was 48.43 months in the AHR high expression group and 136.2 months in the AHR low expression group (*HR* = 1.79, *P*= 0.019). The median overall survival time was 21.27 months in the CYP1A1 high expression group and 39.53 months in the low expression group (Figure 6b). The median overall survival time was 103.23 months in the ESR1 high expression group and 69.8 months in the ESR1 low expression group (Figure 6j). The median overall survival time was 31.83 months in HSP90AA1 high expression group and 37.27 months in the low expression group (Figure 6d), and 45.73 months in the HSP90AB1 high expression group and 136.2 months in the low expression group (Figure 6h). Generally, high expression of AHR, CYP1A1, HSP90AA1, and HSP90AB1 and low expression of ESR1 were not conducive to the prognosis of cervical cancer patients (Figure 7). Analysis of AHR, CYP1A1, HSP90AA1, HSP90AB1 and ESR1 using the Cox multivariate regression model and the forward stepwise regression method (inclusion: *P* < 0.05, exclusion: *P*> 0.1) showed that high expression of AHR (*HR *= 1.874, *95% CI *= 1.069–3.285, *P*= 0.028) and CYP1A1 (*HR *= 1.822, *95%CI *= 1.077–3.080, *P*= 0.025) was a risk factor for the prognosis of patients with cervical cancer (Table 3, Figure 7).

**Table 3.** Multivariate Cox regression analysis

Expression	*B*	*SE*	*Wald*	*HR*	*95%CI*	*P*
AHR	0.628	0.286	4.812	1.874	1.069–3.285	0.028
CYP1A1	0.6	0.268	5.007	1.822	1.077–3.080	0.025

## Immunohistochemistry

**Figure 8.**
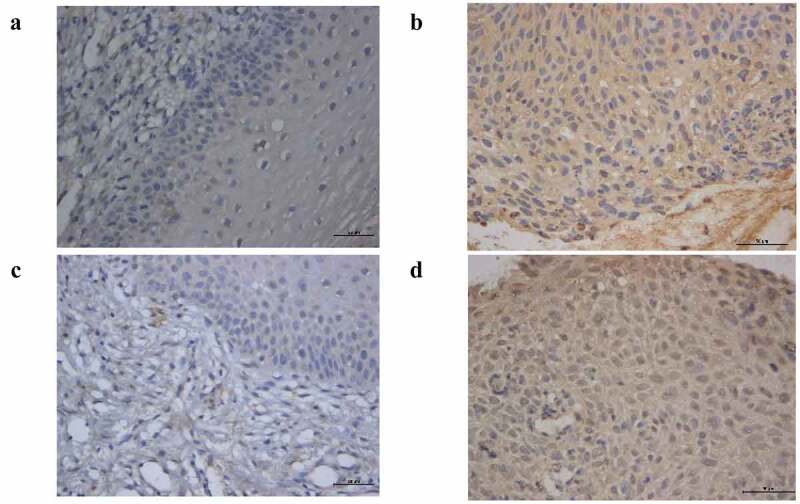
The expression of AHR in normal (a), cancer (b). The expression of CYP1A1 in normal (c), cancer (d)

The expression of AHR and CYP1A1 were analyzed by immunohistochemistry in 30 normal cervical tissue samples and 30 cervical cancer tissue samples. As shown in Figure 8, compared with the normal cervical group, AHR and CYP1A1 are widely expressed in cervical cancer tissues. We found that AHR and CYP1A1 were mainly expressed in cancer cells and immune cells, such as macrophages, and expressed in the nucleus and the cytoplasm. The positive expression of AHR and CYP1A1 differed significantly (*P* < 0.01) between the normal and cancer groups (Table 4).

**Table 4.** The positive expression of AHR and CYP1A1 in cervical tissue

Genes	Normal(n = 30)	Cancer(n = 30)	*F*	*P*
AHR	22.53 ± 16.47	50.22 ± 20.99	26.93	<0.001
CYP1A1	16.51 ± 7.31	38.07 ± 21.03	20.04	<0.001

## Discussion

At present, cervical cancer ranks the fourth leading cause of cancer incidence and death among women in the world [20]. At the time of initial diagnosis, 13% of women with cervical cancer were in advanced stage, which reduced the survival rate of patients. The 5-year disease-specific survival rates of cervical cancer patients was 12% [21]. Many epidemiological evidences support that unmarried status, diseases stage, age at diagnosis, FIGO stage, metastasis, histology grade, treatment method, race, and lung, liver, brain, lymph node metastasis of cervical cancer patients were all associated with prognosis [22,23]. Therefore, it is still a necessary to study the potential mechanism and prognostic biomarkers of cervical cancer.

AHR, a member of the basic Helix-Loop-Helix/Per-Arnt-Sim protein family, is a ligand-activated transcription factor, that is involved in cell proliferation, differentiation, apoptosis, immune regulation and other life processes. AHR is an important regulator of environmental pollutants to modulate their toxic and carcinogenic effects [15]. The roles of AHR in cancer were analyzed in several previous studies. AHR may act as a tumor suppressor in pituitary adenomas [16]. AHR activation by tryptophan catabolites promotes tumor malignancy and suppresses anti-tumor immunity, thereby promoting tumor progression [17]. AHR inhibits redox homeostasis and modulates the tumor promoting microenvironment in breast cancer [18]. The activation of AHR and CYP1A1 and CYP1B1 related pathways promotes the proliferation and migration of gastric cancer cells. Epstein Barr virus can activate the phosphorylation of extracellular signal-regulated kinases in gastric cancer cells by encoding LMP2A. Thus, the functional pathway of AHR is regulated leading to the promotion of gastric cancer development [19]. In female rats, dioxin containing AHR agonist induces, the occurrence of breast cancer and uterine tumors [20], suggesting that AHR is related to the development of cervical cancer. However, the role of AHR in cervical cancer has not been fully elucidated to date.

In this study, we identified 5,860 genes associated with AHR and involved in the development of cervical cancer(*P*< 0.05, FDR < 0.05). Gene set enrichment analysis showed that AHR was involved in a variety of biological regulation processes related to the progression of cervical cancer. With the exception of AIP, AHR and related genes were differentially expressed between cervical cancer and normal tissues was observed in at least one GEO dataset. This indicated a potential interaction between AHR and related genes in cervical cancer. Recent studies indicate that AHR interacts with Estrogen Receptor alpha, thereby changing its conformation, which causes Estrogen Receptor alpha, transcription factors, and multiple protein complexes to stimulate the progress of cervical cancer [21]. In addition, Estrogen Receptor alpha agonists can regulate the activation of AHR pathways in vitro and in vivo [22]. For instance, Estrogen Receptor alpha agonists inhibit the activation of AHR in MCF-breast cancer cells [23]. The AHR-ERS1 pathway promotes the development of cervical cancer. Heterodimer transcription factors comprising AHR and ARNT upregulate the expression of HSP90AA1, AHR, ARNT, SOD1, and SOD2 in correlation with the degree of oxidative stress, suggesting that AHR-ARNT are associated with cell damage [24]. Beischlag [25] et al. verified the protein interaction of ERα-AHR-ARNT in breast cancer cells, which mediated the estradiol-dependent repression of dioxin-induced gene transcription. Epidemiological studies suggest that activation of the AHR-CYP1A1 pathway is related to increased susceptibility in cervical cancer [26]. The increasing expression of CYP1A1 and CYP1B1 is related to the AHR pathway and causes the production of electrophilic derivatives by cytochrome P450 metabolizing enzymes to form DNA adducts, resulting in the activation of oncogenes and inactivation of tumor suppressor genes [27]. These studies together with the findings of the present study provide insight into the potential role of AHR and related genes in cervical cancer.

Increasing evidence supports the role of the tumor microenvironment in cancers, and tumor metastasis and prognosis depend on the level of tumor infiltrating immune cells to some extent [28]. In this study, we examined the correlation between the expression of AHR and related genes and the immune response in cervical cancer using the TIMER and TISIDB databases. The results showed that the expression of CYP1A1, CYP1B1, AIP, ESR1, and MAF was significantly correlated with immune cells, immunoinhibitors, immunostimulators and MHC molecules. In addition, Kaplan-Meier plotter showed that high expression of AHR, CYP1A1, HSP90AA1, and HSP90AB1 and low expression of ESR1 were associated with a high hazard ratio for poor overall survival in cervical cancers. The Cox multivariate regression results further indicated that high expression of AHR and CYP1A1 was a risk factor for the prognosis of patients with cervical cancer. Increased activity of the AHR- IDO1-TDO2 pathway in the tumor microenvironment is related to poor prognosis in Merkel cell carcinoma [29], which is consistent with the results of the present study. Moreover, AHR is a differentiation regulator of T cells. AHR combined with transcription factor c-Maf, promote the trans activation of the IL-10 and IL-21 promoters and the production of Tr1 [30]. Zhang et al. [31] demonstrated that AHR mediated the differentiation of infiltrating T cells in colon cancer, thereby increasing the risk of colon cancer.

In this study, we showed that the expression of CYP1A1, AIP, CYP1B1, ESR1, and MAF was significantly correlated with the level of T cell infiltration in cervical cancer. Moreover, the expression of CYP1A1 was negatively correlated with the level of T cells in cervical cancer, and high expression of CYP1A1 was not conducive to the prognosis of patients with cervical cancer. The expression of ESR1 was positively correlated with the level of T cells, and high expression of ESR1 associated with a favorable prognosis. These results indicated that CYP1A1 and ESR1 were not only potential biomarkers to predict the prognosis of cervical cancer patients, but may also serve as immune-related markers in cervical cancer. The potential of CYP1A1 and ESR1 for predicting prognosis and immune responses in other tumors was reported previously. Vasilis et al. [32] demonstrated that overexpression of CYP1B1 and CYP1A1 in colon and bladder cancer adversely affected the prognosis of patients, which is consistent with the results of the present study. In addition, Zhou et al. [33] suggested that gene polymorphisms of CYP1A1 improved the accuracy of prognosis in metastatic breast cancer, demonstrating the predictive potential of CYP1A1 for tumor therapy and prognosis. An increasing number of studies support the association between ESR1 and tumor immune defense [34]. Increased expression of ESR1 is related to enhanced innate immunity, which inhibits the metastasis of lung cancer [35]. Moreover, the activation of B cells is regulated by ESR1 signaling to some extent in different immune response periods [36–45]. In this study, we found that the level of B cells infiltrated in cervical tumor was positively correlated with high expression of ESR1. This confirmed that the expression of ESR1 enhanced the immunity of the tumor microenvironment of cervical cancer, which may explain its role in the favorable prognosis of patients.

## Conclusion

In conclusion, AHR and related genes were closely associated with cervical cancer. In particular, AHR, CYP1A1 and ESR1 were identified as potential prognostic biomarkers and shown to be correlated with immune responses in cervical cancer. This study provided useful information for the potential clinical diagnosis of cervical cancer, as well as potential molecular markers and immune targets in cervical cancer.

## Supplementary Material

Supplemental Material

## Appreciation

This work was supported by the National Natural Science Foundation of China (No.81760596) and Natural Science Foundation of Xinjiang Uygur Autonomous Region (No.2019D01C209)
